# More Reasons to Move: Exercise in the Treatment of Alcohol Use Disorders

**DOI:** 10.3389/fpsyt.2017.00160

**Published:** 2017-08-28

**Authors:** Mats Hallgren, Davy Vancampfort, Felipe Schuch, Andreas Lundin, Brendon Stubbs

**Affiliations:** ^1^Department of Public Health Sciences, Karolinska Institutet, Stockholm, Sweden; ^2^Department of Rehabilitation Sciences, University of Leuven, Leuven, Belgium; ^3^Centro Universitário La Salle (Unilasalle) Canoas, Canoas, Brazil; ^4^Department of Health Service and Population Research, King’s College London, Institute of Psychiatry, Psychology and Neuroscience (IoPPN), London, United Kingdom; ^5^Physiotherapy Department, South London and Maudsley NHS Foundation Trust, London, United Kingdom

**Keywords:** physical exercise, alcohol use disorders, physical activity, alcohol dependence, substance abuse treatment

Physical exercise is widely regarded as “medicine” for the prevention and treatment of myriad somatic health conditions. Studies also demonstrate that exercise is effective in the treatment of common mental disorders and has a role in the treatment of serious mental illness. Despite this evidence, the benefits of exercise for people with alcohol use disorders (AUDs) remain understudied. This Opinion article presents a rationale for adopting physical exercise interventions in those with AUDs and identifies five key areas of research that warrant further investigation.

## AUDs: Highly Prevalent but Undertreated

Problem drinking that becomes severe is often given the medical diagnosis of AUD. Common features include an inability to control the amount of alcohol consumed, consumption that results in social or work-related problems, and tolerance or the need to drink increasing amounts of alcohol to obtain a desired effect (1). A recent national epidemiologic study in the United States found that the 12-month and lifetime prevalences of AUD were 13.9 and 29.1%, respectively (2), which is comparable with other major health problems such as depression and diabetes (3). The personal and societal impact of AUDs are immense, yet most people with the disorder never seek or receive treatment (4, 5). Several investigations have found that only a minority of alcohol-dependent individuals, around 20% in many studies, receive treatment (4, 6, 7). One explanation for poor help seeking is that AUDs are particularly stigmatized (8). Another issue concerns the range of treatments available, which currently include cognitive behavioral interventions (CBT), motivational interviewing, 12-step facilitation treatment and pharmacological therapy (9). These interventions are effective for some individuals (10), but may not be suitable for all treatment seekers, especially those presenting with milder forms of AUD. This is relevant as approximately 75% of people diagnosed with an AUD experience a mild-to-moderate level of dependence (2, 11). Broadening the array of effective, “lifestyle” focused treatments could increase help seeking and potentially improve adherence (12).

## Effective Treatments for Somatic and Psychiatric Comorbidity are Needed

People with AUDs experience an excess mortality rate two times higher than those without AUDs (13). Compared with the general population, those with AUDs in treatment have a 10-fold risk of mortality from liver cirrhosis and mental disorders, a 7-fold risk for injury fatalities, and a 2-fold risk for cardiovascular and cancer deaths (14). AUDs are also associated with a high prevalence of the metabolic syndrome (MetS) highlighting the generally poor health status of this population (15). Of importance, regular exercise has been shown to significantly reduce the risk of developing MetS and is frequently prescribed for the management of related disorders, including cardiovascular disease and diabetes (16). Psychiatric comorbidity is also highly prevalent in AUDs, and depression has been linked to the etiology of AUD (17). A recent survey reported that the lifetime odds of having any mood disorder was 1.5 times higher in people with an AUD compared to the general population (2). It has also been suggested that alcohol exposure may cause metabolic changes, which act to increase the risk of mood disorders (18). Thus, treatments which are effective in ameliorating both the physical and psychiatric comorbidities associated with AUDs are highly desirable. Several reviews support the antidepressant effects of exercise (19, 20), and it has been shown that people who are physically active and maintain their cardiorespiratory fitness across the lifespan have a reduced risk of experiencing a depressive episode, in addition to other detrimental health outcomes (21).

## Evidence from Intervention Studies: Positive Effects on Depression, Fitness, and Somatic Health

A systematic review by Giesen et al. including 14 RCTs concluded that exercise may have beneficial effects on key domains of physical functioning, including fitness, basal heart rate, physical activity level, and strength (12). A trend toward a positive effect was observed for alcohol craving and drinking behavior. Exercise interventions were considered safe with no adverse events reported. Another narrative review identified 11 studies of which 6 concluded that exercise may have a positive impact on consumption, abstinence, and the urge to drink (craving) (22). Recently, a meta-analysis reported the pooled treatment effects of exercise in AUDs across multiple health outcomes (23). Of 21 studies reviewed, exercise was shown to have significant positive effects on fitness and depression, and a positive but non-significant trend was observed for changes in anxiety and self-efficacy (23). There was no evidence to suggest that exercise reduced self-reported average daily alcohol consumption, or hazardous/harmful drinking patterns; however, these analyses were limited to only two RCTs. For the change in weekly consumption (*n* = 3 studies), a statistically significant difference was observed favoring exercise (SMD = −0.656, *p* = 0.04), but the difference was no longer significant after adjustment for publication bias.

## Five Key Research Questions

These recent reviews suggest that exercise is a safe and feasible intervention for people with AUDs, with important somatic and psychiatric benefits. However, the evidence base is currently limited to a small number of investigations. To advance this field, additional controlled trials with adequately powered participant samples are needed to address the following research questions:

1. **How physically active (and inactive) are people with AUDs?**

The starting point for any public health intervention is descriptive epidemiological data. Presently, little is known about the physical activity habits of those seeking treatment for AUDs. In particular, no studies have used objective assessments (i.e., accelerometers, inclinometers) to measure how much physical activity people with AUDs engage in. Also relevant is the objective assessment of sedentary behavior—too much sitting—which has been linked to depression (24) and anxiety (25) in recent studies. In general population surveys, the relationship between alcohol consumption and physical activity is complex (26), with some studies indicating a positive association between the amount of alcohol consumed and self-reported physical activity levels (27). Our clinical experience suggests that many people with AUDs seeking treatment are inactive, but research is needed to document just how much (or little) these individuals move in daily life.

2. **What are the effects of acute exercise on mood, anxiety, and alcohol craving?**

Affective responses to exercise—that is, how people “feel” during and after exercise—are an important predictor of future exercise participation (28). Specifically, positive affective responses to acute exercise are generally associated with greater exercise participation, while negative mood states predict lower participation to exercise regimes (29). Of importance, it is also known that lowered mood states and anxiety frequently precipitate “urges” to drink alcohol, which in turn can trigger the onset of heavy drinking in people with AUD (30, 31). To date, however, only two studies have examined the acute effects of exercise in alcohol dependent individuals. Ussher et al. (32) randomized 20 recently detoxified alcohol dependent patients (mean age = 40 years) to 10 min of light aerobic exercise (control), and moderately intense aerobic exercise, respectively. Changes in the urge to drink were assessed before, during, and after exercise. Relative to baseline, there was a significant decline in alcohol urges for the experimental condition during (but not after) exercise (32). Another study repeatedly examined the acute effects of moderate exercise on mood, anxiety, and alcohol cravings over a 12-week period in sedentary alcohol-dependent adults (*n* = 26) (33). Improvements in mood and reductions in anxiety and craving were observed at every pre–post-exercise session (33). While these two studies are promising, little is currently known about the acute effects of different modes and intensities of exercise on mood, anxiety, and alcohol craving in alcohol-dependent individuals.

3. **What mechanisms underlie the positive effects of exercise in AUDs?**

As depression is often comorbid with AUDs, and alcohol itself modulates the immune system, a plausible mechanism concerns inflammation. Studies have shown that pro-inflammatory cytokines (e.g., IL-6) and tumor necrosis factor α (TNF-α) play a causal role in the development of depressive-like behavior (34). A study of exercise for depression found a relationship between high baseline levels of IL-6 and greater reductions in posttreatment depression severity (35). Authors also found that reductions in IL-6 were correlated with lower depressive symptoms. As heavy alcohol use is generally associated with elevated pro-inflammatory markers (36), regular physical exercise could reduce systemic inflammation in AUD patients, leading to improved mood states, lower anxiety, and stress-reactivity. An emerging literature based on neuroimaging studies demonstrates that exercise and commonly misused substances activate similar reward pathways in the brain (37–39). Exercise increases the concentration of neurotransmitters, which contribute to exercise-induced reward, and a meta-analysis shows that exercise increases BDNF more than control conditions (40). Exercise also evokes hippocampal neurogenesis (41), a process that may positively affect stress-related disorders, and possibly alcohol dependence. While these biological explanations are plausible, they remain speculative and have not been examined in the context of exercise treatment for AUDs.

4. **Can exercise improve cognitive functioning in AUDs?**

Related to the previous question is whether exercise can improve cognitive functioning in AUDs. A meta-analysis examining cognition in AUDs (*n* = 62 studies) found significant impairments across multiple cognitive functions, including speed of processing, visuospatial ability, executive functioning, learning, and verbal fluency (42). However, the mechanisms linking alcohol use with cognitive impairment are not fully understood. One hypothesis suggests that the prefrontal cortex is particularly vulnerable to the effect of alcohol; an idea supported by neuroimaging studies showing decreased metabolic rates in prefrontal regions in correlation with executive function deficits (43). Another model suggests that long-term heavy drinking leads to a mild generalized dysfunction of the brain, resulting in a variable pattern of impairment (44). Evidence from both human and animal studies suggests that physical activity facilitates neuroplasticity of certain brain structures and may improve cognitive functioning (45). While “moderate” alcohol consumption has been linked in some studies to improvements in cognition, primarily in older adults (46), heavy drinking can have profound negative effects on cognition (47). Such deficits are postulated to elevate the risk of hazardous drinking by increasing attentional biases toward alcohol (48). Recent studies have shown that acute bouts of exercise can reduce cigarette cravings and attenuate smoking-related attentional biases (49, 50), processes that could also apply to alcohol consumption. Previous studies have demonstrated positive associations between exercise and cognitive functioning in adult populations of varying age and health status (45), and among smokers (49); yet, these relationships have not been examined in AUDs.

5. **Sticking with it: what factors influence exercise adherence in this population?**

For exercise interventions to be effective, participants need to “stick with it.” Poor treatment adherence is associated with higher risk of relapse, a worse long-term prognosis, and greater resource utilization (51). A meta-analysis reported a pooled dropout rate from exercise intervention studies for AUDs of 40.3% (23). This is substantially higher than the pooled dropout rate reported from exercise interventions in schizophrenia (26.7%) (52) and depression (18.1%) (53). Of relevance, each of the abovementioned reviews found that supervision of exercise by a suitably qualified trainer was associated with significantly lower dropout rates. However, the varied dropout rates between different diagnostic groups also suggest that factors other than supervision are important, such as the inclusion of a motivational component (53). Currently, no studies have explored the influence of exercise intensity on adherence, and no study has compared the effects of different types of exercise on adherence in this patient group. Identifying factors related to exercise adherence is important for the planning of exercise interventions and will likely improve their effectiveness.

## Conclusion

Alcohol use disorders are highly prevalent, highly comorbid, and often go untreated. Current treatment options are effective for some but not all patients and relapse is common. Physical exercise is a safe and feasible treatment option that can improve comorbid health problems and may reduce heavy drinking. We preface this conclusion with a caveat; the evidence base is currently limited to a small number of studies of varying methodological quality (23). Additional trials are needed to strengthen the case for prescribing exercise to reduce alcohol consumption *per se*. Although research questions remain, we suggest that the high prevalence of multimorbidity in AUDs is a sufficient reason to recommend that physical activity habits should be routinely screened in clinical practice and that exercise be considered as an adjunct treatment in this population.

## Author Contributions

MH initiated the article and wrote the first draft. All coauthors have read and made substantive contributions to the paper.

## Conflict of Interest Statement

The authors declare that the research was conducted in the absence of any commercial or financial relationships that could be construed as a potential conflict of interest.
